# Lambda Red-mediated Recombineering in the Attaching and Effacing Pathogen *Escherichia albertii*

**DOI:** 10.1186/s12575-015-0032-8

**Published:** 2016-02-03

**Authors:** Marisa Egan, Jasmine Ramirez, Christian Xander, Chirag Upreti, Shantanu Bhatt

**Affiliations:** Department of Biology, Saint Joseph’s University, 5600 City Avenue, Philadelphia, PA 19131 USA; Department of Mathematics, Saint Joseph’s University, 5600 City Avenue, Philadelphia, PA 19131 USA; Howard Hughes Medical Institute, Columbia University Medical Center, Columbia, USA; Columbia University Medical Center, 1051 Riverside Drive, New York, NY 10032 USA; Present address: Microbiology Department, Perelman School of Medicine, University of Pennsylvania, 3610 Hamilton Walk, 221 Johnson Pavilion, Philadelphia, PA 19104 USA; Present address: Bluemle Life Sciences Building, Thomas Jefferson University, 233 South Tenth Street, Philadelphia, PA 19107 USA

**Keywords:** Exo, Bet, Gam, Recombineering, LEE, *Escherichia albertii*

## Abstract

**Background:**

The ability to introduce site-specific mutations in bacterial pathogens is essential towards understanding their molecular mechanisms of pathogenicity. This has been greatly facilitated by the genetic engineering technique of recombineering. In recombineering, linear double- or single-stranded DNA molecules with two terminal homology arms are electroporated into hyperrecombinogenic bacteria that express a phage-encoded recombinase. The recombinase catalyzes the replacement of the endogenous allele with the exogenous allele to generate selectable or screenable recombinants. In particular, lambda red recombinase has been instrumental in engineering mutations to characterize the virulence arsenal of the attaching and effacing (A/E) pathogens enteropathogenic *Escherichia coli* (EPEC), enterohemorrhagic *E. coli* (EHEC), and *Citrobacter rodentium. Escherichia albertii* is another member of this taxon; however, the virulence of *E. albertii* remains cryptic despite accumulating evidence that *E. albertii* is an emerging pathogen. Multiple retrospective studies have reported that a substantial number of EPEC and EHEC isolates (~15 %) that were previously incriminated in human outbreaks actually belong to the *E. albertii* lineage. Thus, there is increased urgency to reliably identify and rapidly engineer mutations in *E. albertii* to systematically characterize its virulence determinants. To the best of our knowledge not a single chromosomal gene has been altered by targeted mutagenesis in *E. albertii* since it was first isolated almost 25 years ago. This is disconcerting because an *E. albertii* outbreak could cause significant morbidity and mortality owing to our inadequate understanding of its virulence program.

**Results:**

In this report we describe a modified lambda red recombineering protocol to mutagenize *E. albertii*. As proof of principle, we successfully deleted three distinct virulence-associated genetic loci – *ler*, *grlRA*, and *hfq* – and replaced each wild type allele by a mutant allele with an encodable drug resistance cassette bracketed by FRT sites. Subsequently, the FRT-site flanked drug resistance marker was evicted by FLP-dependent site-specific recombination to generate excisants containing a solitary FRT site.

**Conclusions:**

Our protocol will enable researchers to construct marked and unmarked genome-wide mutations in *E. albertii,* which, in turn, will illuminate its molecular mechanisms of pathogenicity and aid in developing appropriate preventative and therapeutic approaches to combat *E. albertii* outbreaks.

## Background

Whole genome sequencing has ushered in a new era that has revolutionized the field of microbial genetics [1, 2]. Within the past decade sequencing has become more robust, inexpensive, and quick. A scientific sub-discipline that has benefited immensely from rapid genome sequencing is bacterial pathogenesis [3, 4]. The availability of complete genome sequences has enabled bacteriologists to undertake a global investigation of a pathogen’s transcriptome [5–8], proteome [9–11], and metabolome [12, 13] at an unprecedented level. However, the accumulation of such large-scale genome-wide data has left scientists with even more questions than answers [1, 2]. For instance, in any sequenced microbial genome a substantial number of genes remain functionally unannotated or classified as “putative” or “hypothetical” [1, 2]. Similarly, transcriptomic, proteomic, and metabolomic studies, while highlighting the number and nature of specific biomolecules expressed under specific conditions, do not reveal their function [1]. Thus, now, more than ever before, the tools and techniques used in forward (starting from a biological process and determining its genetic basis) and reverse genetics (starting from a gene and determining the biological process(es) controlled by it) will become invaluable and essential towards understanding the functions of genes on a genome-wide scale.

The single most important technique in the molecular toolkit of a microbial geneticist is mutagenesis. Mutagenesis enables a researcher to construct mutations – stable changes in the nucleotide sequence of an organism that are inheritable. Analysis of mutants, in turn, permits researchers to characterize the environmental and regulatory controls of a gene [14, 15], deduce the structure-function relationship of the gene product [16, 17], and determine the regulon and range of biological processes controlled by it [18–20]. Mutations can be classified on the basis of the effect that they have on the expression and/or activity of the gene product. The different kinds of mutations include amorphic (complete loss in expression and/or activity of the gene product), hypomorphic (reduced expression and/or activity), hypermorphic (increased expression and/or activity), neomorphic (a novel function or pattern of expression associated with the gene product), and antimorphic (dominant negative) mutations. To introduce such mutations in bacterial genomes geneticists have developed a diverse range of chemical, biological, and physical mutagens [1]. Some of these mutagens such as base analogues, alkylating agents, radiations, and transposable elements introduce nonspecific and genome-wide random mutations [1, 2]. By contrast, the construction of gene-specific mutations is primarily accomplished by the use of suicide vectors [2, 21, 22] or recombineering [23–32].

A suicide vector is a plasmid that possesses certain key characteristics: a conditional origin of replication that enables the plasmid to replicate under permissive conditions but not under non-permissive conditions, a selectable maker, which confers a phenotypic benefit to the bacterium under specific conditions (e.g. drug resistance or prototrophy), a counterselectable marker, which promotes bacterial death under certain conditions (e.g. an enzyme that converts a pro-drug to a drug toxic to the bacterium), and a polylinker region to facilitate cloning [2, 21, 22, 33]. The desired mutant allele is inserted into the polylinker region and the recombinant vector is introduced into a suitable bacterial transcipient. The transcipient is subsequently grown under conditions that are not only non-permissive for plasmid replication but also select for bacteria that stably express the plasmid-encoded selectable trait. The mutant colonies that are observed are merodiploid integrants, in which the plasmid encoded mutant allele has undergone homologous recombination with the chromosomally encoded wild type allele thereby inserting the entire plasmid into the bacterial chromosome and facilitating the expression of the selectable marker. Integrants can then be grown under conditions where the counterselectable marker is expressed to induce toxicity and promote bacterial lethality. Any observed excisants are typically those in which the counterselectable marker, along with the plasmid backbone, has been successfully evicted. This occurs by a second recombinational event between the wild type and mutant allele, which either restores the original wild type allele or replaces it with the mutant allele [2, 21, 22]. Suicide vectors can be used to introduce a gamut of precise chromosomal modifications in different bacteria [34–38]. However, in today’s day and age of genetic engineering the reliance on suicide vectors to introduce gene-specific mutations has diminished substantially. This is because this technique is cumbersome, requires prior cloning of the mutant allele onto the suicide vector, is limited by the presence of restriction sites on the plasmid, prone to illegitimate recombinational events, and logistically may span several days.

Within the past decade the revolutionary technique of recombineering has largely replaced the use of suicide vectors to construct locus specific mutations [23, 25–28, 31, 32, 39–41]. Recombineering circumvents the need to rely on restriction enzymes, DNA ligases, and cloning into suicide vectors for allelic replacement. Recombineering, a portmanteau for *recombi*nation-mediated genetic engi*neering*, is a technique for manipulating the bacterial genome in vivo by using phage-encoded recombinases [31]*.* The most frequently employed recombinases include the Rac prophage-encode RecET [32, 42–44] and the lambda prophage-encoded Red [23–28, 31, 41]. Bacteria that express RecET or lambda Red proteins are rendered hyperrecombinogenic and are capable of catalyzing efficient homologous recombination between linear single-stranded (ss) or double-stranded (ds) DNA molecules with two short terminal arms that share ~40–50 nucleotides of homology to a target DNA [23–26, 32, 40, 45]. The RecET system consists of two proteins – RecE and RecT [32]. RecE is a ds specific DNA exonuclease that sequentially degrades the 5′ end of DNA to generate single stranded (ss) 3′ overhangs [42]. RecT, in turn, binds to the ssDNA and facilitates base pairing between the two homology arms and complementary sequences on the target DNA resulting in homologous recombination and the eventual replacement of the chromosomal allele with the mutant allele [32, 44]. The lambda Red recombination system includes three proteins – Exo, Beta, and Gam [23, 26, 27, 31, 41]. Exo, like RecE, is a 5′→3′ dsDNA specific exonuclease that generates a ssDNA intermediate [39, 46]. Beta, like RecT, binds to the ssDNA intermediate and base pairs it to its complementary single stranded target to form a heteroduplex and facilitate homologous recombination [47, 48]. An accessory component of the lambda red system is Gam. Gam inhibits the RecBCD and SbcCD nucleases thereby preventing the degradation of dsDNA to enhance recombination [49, 50]. When linear dsDNA is used as a substrate then both the exonuclease (RecE/Exo) as well as the ssDNA binding protein (RecT/Bet) are necessary for recombineering [29, 51]. By contrast, when ssDNA/oligonucleotides are used, then the ssDNA anealing protein is sufficient for homologous recombination [40, 52, 53]. Furthermore, recombination frequencies are orders of magnitude higher using ssDNA compared to dsDNA [29].

The lambda Red recombineering system, in particular, has transformed our understanding of the molecular mechanisms of bacterial pathogenicity in a wide range of Gram-negative pathogens including several members of the attaching and effacing (A/E) family of pathogens, which encompasses enteropathogenic *Escherichia coli* (EPEC), enterohemorrhagic *E. coli* (EHEC), *Citrobacter rodentium*, and *Escherichia albertii* [14, 15, 25, 35, 37, 54]*.* A/E pathogens are classified as such because upon infection the bacterium depolymerizes actin filaments, thereby destroying the structural and functional integrity of the intestinal microvilli (effacement) [14, 15, 55, 56]. Thereafter, the depolymerized actin is recruited beneath the adherent bacterium and repolymerized to form actin-filled membrane-enclosed evaginations called pedestals that are capped by the infecting bacterium (attachment) [14, 15, 56, 57]. The overall pathological manifestation of attachment and effacement is the diminished ability of enterocytes to absorb water and nutrients, which leads to diarrhea [14, 15].

The pathogenicity island locus of enterocyte effacement (LEE) is essential for pedestal formation [14, 15, 58–61]. The LEE encodes a functional type 3-secretion system (T3SS) that punctures the host cell membrane and allows A/E pathogens to inject a range of effector molecules directly into the infected cell [37, 62–69]. These effector molecules are involved in the subversion of host signal transduction and regulatory pathways, which lead to the observed ultrastructural changes including microvillar disintegration and pedestal formation – diagnostic hallmarks of infection by the A/E pathogens [14, 15]. Thus, understanding the regulation of the LEE is imperative to develop efficacious prophylactic and/or therapeutic measures. Between EPEC and EHEC virtually every conceivable mutation – point, substitutions, insertions, deletions, epitope tagging, and marked– has been engineered by recombineering [25, 35, 70–72]. This constellation of mutations has revealed an intricate regulatory landscape that fine-tunes gene expression from the LEE in response to diverse environmental stimuli [14, 15, 73]. Moreover, recombineered EPEC and EHEC mutants have also yielded mechanistic insight into several *trans-*acting factors and *cis-*acting elements [14, 15, 74]. Similarly, in the murine pathogen *Citrobacter rodentium* recombineering was used, in part, to systematically mutate every gene that is encoded within the LEE to elucidate the genetic control of pathogenesis [37]. This study was groundbreaking in that the researchers identified two novel LEE-encoded regulators, GrlR and GrlA, that repress and activate the LEE respectively [37]. Besides GrlR and GrlA, the LEE also encodes the master transcriptional regulator, Ler [75, 76]. Ler, GrlR, and GrlA are conserved between *Citrobacter rodentium*, EPEC, and EHEC [14, 15, 37, 73, 75, 76]. These transcription factors are indispensible for the synchronized spatiotemporal regulation of genes expression from the LEE, which climaxes with pedestal morphogenesis and the accompanying virulence of A/E pathogens [14, 15]. Intriguingly, even though the lambda red proteins are operational in A/E pathogens, induction of a hyperrecombinogenic state depends upon the vector that expresses these genes. For instance, Murphy and Campellone observed that EHEC recombinants are readily obtainable only when the lambda red genes were expressed from the low-copy plasmids pKM201 or pKM208 but not when the expression platform was the high-copy plasmid pTP223 [25]. Reciprocally, in EPEC, recombinants were only observed when the lambda red proteins were expressed from pTP223 [25]. Similar observations have also been reported in *E. coli* [23]*.* Thus, the expression of lambda red proteins from a suitable vector is instrumental in engineering defined mutations in bacteria.

Whereas recombineering has been effectively utilized to understand the molecular pathogenesis of EPEC, EHEC, and *Citrobacter rodentium*, it has, thus far, not been adapted for *Escherichia albertii* [25, 35, 37, 54, 77, 78]. *E. albertii* was first isolated from diarrheal stool samples of five children from Bangladesh by John Albert and his collaborators at the International Centre for Diarrheal Disease Research, Bangladesh (ICDDR-B) in the early 1990s [79]. The children were febrile, presenting with watery diarrhea, dehydration, vomiting, and abdominal distension. Initially, these isolates were classified as *Hafnia alvei* [79]. However, Albert et al. subsequently discovered that these isolates possessed the *eae* gene, which is normally present in A/E pathogens but absent in *H. alvei. eae* codes for intimin – an outer membrane protein essential for pedestal formation by A/E pathogens [80]. Moreover, a comparison of the 16S rRNA sequence of these strains with *eae-*negative *H. alvei* isolates revealed a low degree of sequence similarity, thereby warranting their taxonomic reclassification [81]. Subsequent attempts to accurately re-classify these diarrheal isolates also yielded ambiguous results [82]. The same isolates yielded highly variable phenotypic and biochemical profiles characteristic of *Yersinia ruckeri*, *Salmonella enterica*, *H. alvei*, EHEC, and *Citrobacter* depending upon the commercial diagnostic kit used for taxonomic classification [82]. More recently though, using a battery of conventional biochemical and phenotypic assays, 16S rRNA sequencing, DNA-DNA hybridization, and allele-specific PCR these isolates were unambiguously shown to be distantly related to the *Escherichia* (55–64 %) and *Shigella* (54–60 %) genera and have been defined as a novel *Escherichia* species, *Escherichia albertii* [82, 83]. Still, very little is known about this diarrheal pathogen today, so its misidentification persists. A recent retrospective study re-examined 179 clinical strains that were originally isolated from diseased humans and animals and had been classified as EPEC or EHEC on the basis of routine diagnostic assays. Multilocus sequencing of these strains revealed that of these strains, 26 (14.5 %) were actually *E. albertii* isolates that had been incorrectly assigned to the EPEC or EHEC taxa [84]. Similarly, EPEC was implicated as the etiologic agent for a foodborne outbreak in Fukuoka and Kumamoto cities of Japan in 2003 and 2011 respectively. However, reexamination of these strains by PCR and additional biochemical assays confirmed them to belong to the *E. albertii* lineage [85, 86]. In another recent study an *E. albertii* strain possessing the *stx*_2a_ allele was isolated from a patient who suffered from bloody diarrhea in Norway [87]. Shiga toxin 2a (Stx2a) is one of the most potent isoforms of the Shiga toxin that has been implicated in hemorrhagic colitis and the life-threatening complication hemolytic uremic syndrome (HUS) [88–91]. Prior to this report, only the *stx*_2f_ allele, which produces mild symptoms and has no prior link to bloody diarrhea or HUS, was reportedly observed in a subset of *E. albertii* strains [84, 87]. In the genus *Escherichia stx2a* is most frequently associated with EHEC [92], and to a lesser degree with enteroaggregative *E. coli* (EAEC) [93]. This presence of this allelic variant has often been used as a reliable marker to narrow down the *Escherichia* isolate to these pathotypes. However, the presence of *stx*_2a_ in *E. albertii* suggests that some of *stx*_2a_ possessing *E. coli* strains that are currently designated as EHEC or EAEC may actually be *E. albertii.* Moreover, this genotypic feature accentuates the virulence potential of *E. albertii.* Clearly, there is increased urgency to reliably diagnose *E. albertii* isolates and rapidly identify and characterize the repertoire of its virulence determinants, determine their mode of action, and the virulence-associated processes controlled by them – a formidable task that requires the engineering of specific and random *E. albertii* mutants.

While the genome of *E. albertii* has been sequenced, to the best of our knowledge, we have not encountered any reports demonstrating chromosomal modifications in *E. albertii*. Here we describe a simple modified protocol using the lambda Red recombineering system to generate chromosomal mutations in *E. albertii*, which appears to be genetically recalcitrant*.* Using this protocol we successfully deleted the LEE-encoded genes, *ler* and *grlRA*, and the non-LEE encoded gene *hfq*, albeit at frequencies lower than those reported in *E. coli*. This protocol will aid pathogeneticists to rapidly construct knockout, knockin, epitope-tagged, transcriptional, translational, and other subtle mutations in *E. albertii* in order to comprehensively characterize its molecular mechanisms of virulence to successfully counteract this emerging broad host pathogen.

## Results and Discussion

Our initial attempts at recombineering in *E. albertii* using pKD46 were largely unsuccessful or non-reproducible (data not shown). Previous research has shown that recombineering efficiency is significantly enhanced in the related bacterium *E. coli* when the lambda red genes are expressed from the pSIM rather than the pKD family of plasmids [28]. The pSIM family of plasmids is derived from the defective lambda prophage in which the minimal lambda red recombinase operon, consisting of the genes *gam, bet,* and *exo*, is expressed from the *p*_*L*_ promoter under the transcriptional control of the thermolabile CI857 lambda repressor [28]. At low temperatures ranging from 30–34 °C CI857 is active and binds to the operator site thereby sterically hindering the promoter and preventing transcription. A thermal upshift to 42 °C reversibly denatures the CI857 repressor thereby inactivating it and leading to the derepression of the *gam*, *bet*, and *exo* genes thereby inducing a temporary hyperrecombinogenic state in the bacterium [28]. When such a bacterium is transformed with dsDNA or oligonucleotides that possess two terminal arms of ~40–50 nucleotides of homology to a target gene the lambda red recombinase promotes the replacement of the endogenous allele with the exogenous allele. Transcription from *p*_*L*_ promoter can be shut off by transferring the cultures back to 30 °C at which the CI857 repressor renatures and binds to the operator to prevent further transcription. Reversible repression by CI857 prevents the unregulated expression of the lambda red genes, which has previously been shown to be mutagenic [25].

**Fig. 1:**
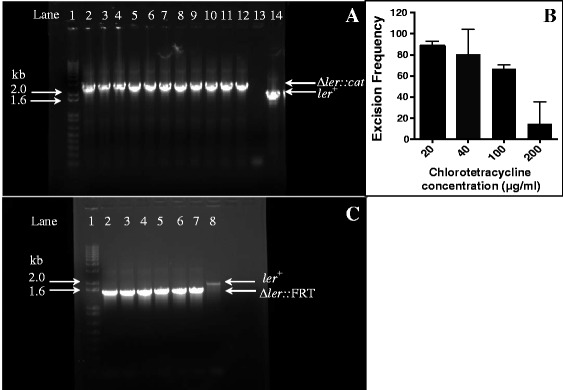
**a** Locus specific PCR to confirm the replacement of *ler* ^*+*^ with ∆*ler::cat* allele in *E. albertii* by recombineering*.* Twelve colonies that arose on chloramphenicol plates were screened by PCR, using a pair of primers, one of which bound upstream (SB2456) and the other downstream (SB2457) relative to the recombination site, to confirm that the Cm^R^ phenotype resulted from the acquisition of the ∆*ler::cat* allele and the concomitant loss of the *ler* ^*+*^ allele. An aliquot (4 μl) of each PCR product was electrophoresed on a 1 % agarose gel and stained with ethidium bromide prior to visualization using a transilluminator (BioRad). All (lanes 2–12), but one (lane 13), isolates had successfully replaced the wild type *ler* ^*+*^ allele (~1.8 kb) with the mutant ∆*ler::cat* allele (~2.5 kb). Lane 1 – 1 kb TrackIt^TM^ Plus DNA Ladder; lanes 2–13 – candidate ∆*ler::cat* recombinants; lane 14 – wild type *ler* ^*+*^ allele. **b** – Eviction of the *cat* cassette from the ∆*ler::cat* locus. Recombinants were transformed with pFT-K, a thermolabile plasmid that expresses the FLP recombinase enzyme under the control of the TetR repressor. Varying concentration of chlorotetracycline (20–200 μg/ml) was added to derepress the *flp* gene and the frequency of excisants was calculated. The excision frequency was highest at the lowest chlorotetracycline concentration and gradually decreased with increasing chlorotetracycline concentration. Results depict the mean ± standard deviation from a representative experiment involving two independently isolated recombinants. At least 36 candidate excisants per ∆*ler::cat* recombinant, or all if fewer arose, were phenotyped to determine the excision frequency. **c** – Locus specific PCR to confirm the eviction of the *cat* cassette from the ∆*ler::cat* recombinants. Excisants that were phenotypically Cm^S^ were screened by PCR using SB2456 & SB2457 to confirm that resensitization to chloramphenicol occurred due to the loss of the *cat* cassette. All isolates that were phenotypically Cm^S^ (lanes 2–7) had evicted the *cat* cassette and gave a PCR product of the expected size (~1.55 kb) compared to the wild type allele (lane 8; ~1.8 kb). Lane 1 – 1 kb TrackIt^TM^ Plus DNA Ladder; lanes 2–7 – candidate ∆*ler::*FRT (Cm^S^) excisants; lane 8 – wild type *ler* ^*+*^ allele

Induction of the lambda red genes for as little as 15 min generates a large number of recombinants in *E. coli*. We used conditions similar to those previously described for *E. coli* to engineer hyperrecombinogenicity in *E. albertii.* LS5504 (*E. albertii* strain Albert 19982^T^ transformed with pSIM6) was thermally induced for 7.5, 15, or 30 min at 42 °C and subsequently made electrocompetent. Electrocompetent cells were then transformed with 1.5 μg of ∆*ler::cat* amplicon at 1.8 kV. After overnight recovery cultures were spread on LB plates supplemented with chloramphenicol (3.125 μg/ml) and screened to identify Cm^R^ recombinants. No recombinants were obtained under these experimental conditions. Interestingly, when the electroporation voltage was increased to 2.5 kV, a reproducible increase in recombineering was observed with ~14.0 ± 1.3 recombinants per 10^10^ cfu per μg of DNA. Recombinants were observed at chloramphenicol concentrations as high as 6.25 μg/ml (data not shown). At higher concentrations (12.5–25 μg/ml) no recombinants were observed. Interestingly, the higher concentrations correspond to the selective conditions typically used by us to isolate recombinants of EPEC and *E. coli* (data not shown)*.* Thereafter, we proceeded to vary other experimental parameters to further enhance recombineering efficiency. We reasoned that prolonged expression of the lambda red genes could further increase recombineering. Consistent with this prediction induction of the red operon for an hour at 42 °C followed by electroporation at 2.5 kV further increased the recombination frequency by ~3.3-fold (46.2 ± 7.3 recombinants per 10^10^ CFU per μg of DNA). From one such experiment 12 Cm^R^ mutants were screened by locus specific PCR to verify that the Cm^R^ phenotype stemmed from the allelic replacement of *ler*^*+*^ with the ∆*ler::cat* allele (Fig. 1a). Of the 12 isolates screened, 11 had successfully replaced the wild type allele with the mutant allele (Fig. 1a). pSIM6 was cured from the recombinants by culturing them at 42 °C.

We then proceeded to test the feasibility of evicting the *cat* cassette that is bracketed by direct repetitive FRT sites. Two such ∆*ler::cat* mutants (LS5568 & LS5570) were transformed with the plasmid pFT-K, which expresses FLP recombinase under the control of the TetR repressor, to generate LS5592 and LS5593 respectively. Unautoclaved chlorotetracycline was added at a range of concentrations (20–200 μg/ml) to inactivate TetR to derepress FLP recombinase. FLP recombinase catalyzes the site-specific recombination between the two FRT sites to evict the *cat* cassette and generate excisants that are phenotypically Cm^S^ and contain a single FRT site. It was observed that the excision frequency decreased precipitously as the concentration of chlorotetracycline increased (Fig. 1b). At a chlorotetracycline concentration of 20 μg/ml ~90 % of the observed colonies had evicted the *cat* cassette, whereas at 200 μg/ml the eviction frequency was reduced to ~15 % (Fig. 1b). This was unexpected because we anticipated that increasing the chlorotetracycline concentration would lead to higher expressivity of *flp* and enhance excision frequency. However, we did observe that as the chlorotetracycline concentration was increased the total CFU count decreased substantially (data not shown). Perhaps, the slowed growth rate may detrimentally affect the expression and/or activity of the FLP recombinase enzyme. Cm^S^ excisants were also verified by locus specific PCR to confirm that the sensitivity to the antibiotic correlated with the loss of the *cat* cassette and the presence of the ∆*ler::*FRT allele (Fig. 1c). pFT-K was successfully cured from the excisants by incubating them at 42 °C overnight and screening for Kan^S^ isolates (data not shown).

**Fig. 2:**
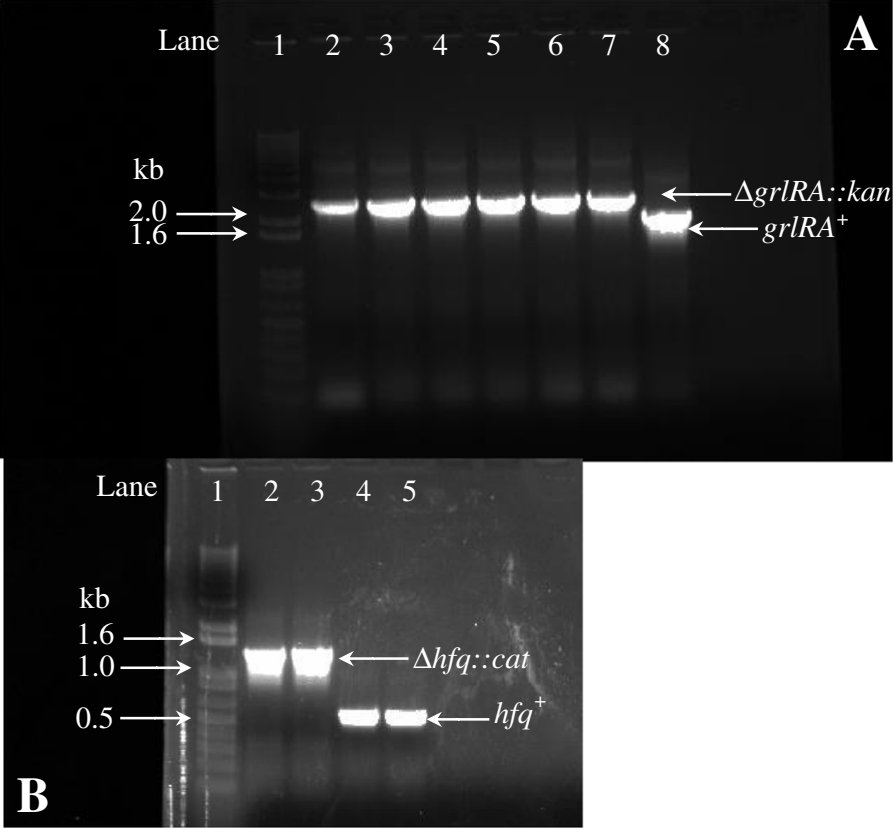
Locus specific PCR to confirm the replacement of *grlRA* ^*+*^ with ∆*grlRA::kan* allele (**a**) and *hfq* ^+^ with ∆*hfq::cat* (**b**) in *E. albertii* by recombineering*.* **a** – Three colonies were selected from LB plates supplemented with kanamycin at 20 and at 22.5 μg/ml and screened by PCR using the primer pair SB2470 and SB2471 to confirm that the Kan^R^ phenotype arose from the acquisition of the ∆*grlRA::kan* cassette and the concomitant loss of the *grlRA* ^*+*^ locus. An aliquot (4 μl) of each PCR reaction was electrophoresed and visualized essentially as described previously. All the Kan^R^ isolates (lanes 2–7) tested positive for the mutant ∆*grlRA::kan* allele (~2.3 kb) and negative for the wild type allele (~1.8 kb). Lane 1 – 1 kb TrackIt^TM^ Plus DNA Ladder; Lanes 2–7 – candidate ∆*grlRA::kan* recombinants isolated on 20 μg/ml (lanes 2–4) or 22.5 μg/ml kanamycin (lanes 5–7). Lane 8 – wild type *grlRA* ^*+*^ allele. **b** – The only colony observed on the Cm plate (6.25 μg/ml) was verified by PCR, using the primer pair SB2485 and SB2486, to confirm the replacement of the *hfq* ^*+*^ allele (~527 bp) with the ∆*hfq::cat* allele (~1.27 kb). Lane 1–1 kb TrackIt^TM^ Plus DNA Ladder; Lanes 2–3 – replicate PCR reactions with the solitary Cm^R^ isolate; Lanes 4–5 – replicate PCR reactions for the wild type *hfq* ^*+*^ allele

The usefulness of lambda red recombineering arises from the fact that it can be utilized to engineer a plethora of mutations at virtually any genetic loci in a range of bacteria [23–26, 37]. Therefore, we next determined if recombineering was equally versatile in *E. albertii*. To address this we attempted to mutate the *grlRA* operon, located within the LEE, and the *hfq* gene, located outside the LEE. The LEE-encoded *grlRA* locus specifies the two transcription factors GrlR and GrlA that exhibit conserved synteny in all the A/E pathogens including *E. albertii* [37, 94–97]. GrlR and GrlA synchronize gene expression from the LEE that culminates with pedestal morphogenesis by EPEC, EHEC, and *Citrobacter rodentium* [14, 15, 37, 70, 98]*.* Meanwhile, Hfq functions as an RNA chaperone that facilitates base-pairing between sRNAs and their target mRNAs to affect RNA stability and/or translation [99, 100]. Hfq has a prominent role in controlling the LEE in EPEC and EHEC [18, 19, 94] (Bhatt S. Unpublished observations). However, the roles of *grlR*, *grlA*, and *hfq* remain unexplored in *E. albertii*. The ∆*grlRA::kan* and ∆*hfq::cat* mutant alleles were synthesized by PCR essentially as described above. The ∆*grlRA::kan* amplicon possesses two terminal homology arms of 50 nucleotides in length. The upstream arm is identical to the region immediately upstream of the *grlR* ORF whereas the downstream arm is identical to the 3′ end of the *grlA* ORF (30 nucleotides) and 20 nucleotides downstream off of it. Thus, upon successful recombineering, the entire *grlRA* operon excluding the terminal 30 nucleotides of the *grlA* ORF and its 3′ UTR are replaced by a *kan* cassette bracketed by FRT sites. The ∆*grlRA::kan* amplicon was electroporated into hyperrecombinogenic bacteria using the optimized recombineering protocol. After recovering the cultures overnight, aliquots were spread onto LB plates supplemented with varying concentrations of kanamycin (12.5–22.5 μg/ml) and screened for Kan^R^ recombinants. Two types of colonial morphologies were readily apparent on kanamycin plates: big sized colonies and punctiform satellite colonies. We observed that at a concentration of 12.5 μg/ml comparable numbers of big colonies were observed for both the transformed and untransformed control (data not shown). Some punctiform colonies were also observed (<20 CFU). However, at higher kanamycin concentrations (17.5–22.5 μg/ml) big colonies were observed only in the case of the transformed cultures (17, 31, and 34 recombinants/μg of DNA on 17.5, 20, and 22.5 μg/ml of kanamycin respectively). Nonetheless, the number of punctiform satellite colonies remained unchanged (<20 CFU) at 17.5 and 20 μg/ml of kanamycin. However, punctiform colonies were rarely observed at 22.5 μg/ml of kanamycin. Three big colonies were randomly picked from the LB plates containing the two highest concentrations of kanamycin (20 and 22.5 μg/ml) and screened by PCR to confirm allelic replacement (Fig. 2a). Lanes 2–4 and 5–7 correspond to the PCR product obtained from potential recombinants that arose on plates containing 20 or 22.5 μg/ml of kanamycin respectively. All the candidate Kan^R^ isolates had successfully replaced the *grlRA* operon with the ∆*grlRA::kan* allele (Fig. 2a, lanes 2–7). Whereas ∆*ler::cat* and ∆*grlRA::kan* recombinants were readily obtained in *E. albertii*, recombination at the *hfq* locus was relatively recalcitrant. No recombinants were observed in our first attempt whereas in the 2^nd^ attempt only 1 Cm^R^ isolate was observed after incubating the plates for 48 h. This isolate was confirmed to have substituted *hfq*^+^ with the ∆*hfq::cat* allele (Fig. 2b, lanes 2–3). Previous reports suggest that the *hfq* mutant in *E. coli*, EHEC, and other phylogenetically related bacteria exhibits a pronounced growth defect and frequently accumulates suppressor mutations [101]. Moreover, the *hfq* mutant is also hypersensitive to diverse stressors including genotoxic agents [101]. It is plausible that *hfq* has an analogous role in *E. albertii*, which may explain the low mutational frequency at the *hfq* locus in the bacterium.

## Conclusion

The ability to reliably and rapidly mutagenize the genomes of bacterial pathogens by recombineering has enabled researchers to have a meticulous understanding of the environmental and regulatory controls of virulence genes, the mechanism of action of virulence factors, and the targets and traits controlled by these virulence factors. In particular, within the last decade the intricacies of pathogenicity accompanying A/E pathogens, such as EPEC, EHEC, and *Citrobacter rodentium* have been unraveled and their arsenal of virulence determinants systematically characterized [14, 15, 25, 37]. This has enabled researchers and health care providers to develop suitable treatments to counteract these pathogens. However, the virulence regulome of the related A/E pathogen *E. albertii* remains cryptic, despite accumulating evidence that this bacterium causes disease in humans and birds and has been classified by the CDC as an emerging pathogen of significant public health concern [84, 102]. To date, not a single genome re-engineering technology has been reported for this bacterium. This is particularly disconcerting because a large-scale outbreak by *E. albertii* may have catastrophic consequences and lead to significant morbidity and mortality owing to our lack of understanding of its prevalence, molecular pathogenesis, and epidemiology.

In this report we have identified a set of conditions for successful lambda red mediated-recombineering of the genome of *E. albertii.* Briefly, hyperrecombinogenicity can be induced when the lambda red genes in pSIM6 are thermally induced at 42 °C for at least an hour prior to making cells electrocompetent. Electrocompetent cells are electroporated at 2.5 kV with linear dsDNA substrate containing short terminal arms of ~50 nucleotide of homology to a target gene. Electroporated cells are recovered overnight at 37 °C/250 rpm for >16 h after which they are plated on selective media to identify candidate recombinants. Whereas these parameters are suitable for reproducible recombineering in *E. albertii*, however, the frequencies are ~ 4 orders of magnitude lower than what has been reported for nonpathogenic *E. coli* [28]. Moreover, consistent with previous observations in EPEC, EHEC, and *E. coli* [23, 25], recombination frequency is greatly affected by the plasmid expressing the lambda red proteins. Recombinants were readily and reproducibly obtained when the lambda red genes were expressed from pSIM6 but not from pKD46. The reason for this observation is unclear. Current efforts in our lab are dedicated towards identifying parameters for further optimizing recombineering in *E. albertii.* Several factors can influence homologous recombination. For instance, increasing the length of the homology arms as well as the amount of DNA can enhance recombination frequencies [26, 52, 103]. Similarly, oligonucleotide/ssDNA-mediated recombineering yields more recombinants than dsDNA-mediated recombineering [28, 29]. Moreover, covalent modification of the 5′ end of ssDNA with a phosphorothioate linkage has been shown to further improve recombination in different bacterial hosts [39]. We are in the process of evaluating some of these parameters to potentiate lambda red mediated recombineering in *E. albertii.*

In conclusion, our formulated protocol for recombineering will enable researchers to construct a plethora of both marked and unmarked mutations in the genome of *E. albertii*. Genetic, biochemical, and phenotypic analysis of these mutants will not only shed light on the regulatory and signal transduction networks that govern virulence gene expression but also on the structure, function, mechanism of action, and regulon of the encodable virulence gene products.

## Methods

### Bacterial Strains, Plasmids, Primers & Media

Bacteria were grown in Luria-Bertani (LB) broth supplemented with appropriate antibiotics when needed. The antibiotics used were chloramphenicol (3.125–25 μg/ml), kanamycin (12.5–25 μg/ml), chlorotetracycline (20–200 μg/ml), and ampicillin (100 μg/ml). Strains and plasmids used in this study are listed in Table 1 whereas oligonucleotides are listed in Table 2. The *E. coli* strain DH5α was used for the propagation and purification of plasmids.

**Table 1:** Bacterial strains and plasmids used in this study

Strain	Relevant genotype, phenotype	Reference/Source
LS5494	*Escherichia albertii* Albert 19982^T^	Manan Sharma
LS5504	LS5494(pSIM6), Amp^R^ (Ts)	This study
LS5568	LS5494∆*ler::cat*, Cm^R^ Clone #1	This study
LS5570	LS5494∆*ler::cat*, Cm^R^ Clone #2	This study
LS5592	LS5568(pFT-K), Cm^R^ Kan^R^ (Ts)	This study
LS5593	LS5570(pFT-K), Cm^R^ Kan^R^ (Ts)	This study
LS5598	LS5494∆*grlRA::kan*, Kan^R^	This study
LS5607	LS5494∆*ler::*FRT = LS5592∆*cat* ∆pFT-K, Cm^S^ Kan^S^	This study
LS5609	LS5494∆*ler::*FRT = LS5593∆*cat* ∆pFT-K, Cm^S^ Kan^S^	This study
LS5612	LS5494∆*hfq::cat*, Cm^R^	This study
DH5α	*supE44 ∆lacU169* (Φ80 *lacZ∆*M15) *hsdR17 recA1 endA1 gryA96 thi-1 relA1*	Bettina Bommarius
Plasmids		
pSIM6	*p*_*L*_ *-gam-bet-exo* genes under the control of CI857 repressor, Amp^R^ (Ts)	Don Court
pKD46	*p*_*araBAD*_ *-gam-bet-exo* under the control of AraC, Amp^R^ (Ts)	Barry Wanner
pKD3	Template plasmid to amplify FRT-*cat-*FRT amplicon, Amp^R^ Cm^R^	Barry Wanner
pKD13	Template plasmid to amplify FRT-*kan-*FRT amplicon, Amp^R^ Kan^R^	Barry Wanner
pFT-K	*flp* gene under the control of Tet^R^ repressor, Kan^R^ (Ts)	Fred Blattner

Tet – Tetracycline, Cm – chloramphenicol, Amp – ampicillin, Kan – Kanamycin, superscripts “R” and “S” denote resistance and sensitivity respectively

**Table 2:** Oligonucleotides used in this study

Primers	Purpose	Sequence
SB2454	5′ primer for replacing *ler* with *cat*	GATTAGGTCATTAATAGCTTAATATATTAAAGCATGCGGAGATTATTTATcatatgaatatcctcctta
SB2455	3′ primer for replacing *ler* with *cat*	TATCGTTATCATCTAATGGTTTTATATTAAATATTTTTCAGCGGCATTAAgtgtaggctggagctgcttc
SB2456	5′ primer to verify *ler* deletion	*gcg* **GAATTC**CCTTTAGCTCCTGGCACTTTTGAAC
SB2457	3′ primer to verify *ler* deletion	*gcg* **AAGCTT**TGACGTAATATTTTATTCAGCTGAT
SB2464	5′ primer for replacing *grlRA* with *kan*	TGGATAGAACAAATTGAAAGGAGTGAGGTTGGTATGAAACTGAGTGAGTTgtgtaggctggagctgcttc
SB2467	3′ primer for replacing *grlRA* with *kan*	ATGTATGTGAAAAGTTATGTCTAACTCCCTTTTTTCCGTCTCATGATCATttccggggatccgtcgacct
SB2470	5′ primer to verify *grlRA* deletion	*gcg* **GAATTC**TGAGCCTGTGGCACAATTGAT
SB2471	3′ primer to verify *grlRA* deletion	*gcg* **AAGCTT**GCAGTCTGATGAAGTGATCCC
SB2481	5′ primer for replacing *hfq* with *cat*	TCAGAATCGAAAGGTTCAAAGTACAAATAAGCATATAAGGAAAAGAGAGAcatatgaatatcctcctta
SB2482	3′ primer for replacing *hfq* with *cat*	AAAAACAGCCCGAAACCTTATTCGGTTTCTTCGCTGTCCTGTTGCGCGGAgtgtaggctggagctgcttc
SB2485	5′ primer to verify *hfq* deletion	*gcg* **GAATTC**GCTATCGCAGGCTGAATGTGT
SB2486	3′ primer to verify *hfq* deletion	*gcg* **AAGCTT**ACCAGCATCATAACGGTCAAA

Uppercase unbolded letters = oligonucleotide sequence homologous to the bacterial chromosome; lowercase letters = sequence complementary to the template pKD3 (for *cat*) or pKD13 (for *kan*); Uppercase bolded letters = Restriction sites; lowercase italicized letters – trinucleotide sequence flanking restriction sites

### Transformation of *E. albertii* with Plasmids Expressing the Lambda Red Enzymes

*E. albertii* strain Albert 19982^T^ (LMG 20976^T^ = CCUG 46494^T^) was streaked onto LB plates and grown overnight at 37 °C. The next day a single colony was inoculated into 5 ml of LB and grown overnight at 37 °C/250 rpm. The following day the overnight culture was subcultured 100-fold into 30 ml of LB and grown to an optical density of ~0.6–0.8. The culture was rendered electrocompetent by multiple washes with ice-cold 10 % glycerol and concentrated to a final volume of 400 μl. Approximately 90 μl of the culture was electroporated with 100 ng of the plasmid pKD46 or pSIM6. The electroporants were recovered in 1 ml of LB and grown for 3 h at 30 °C, after which 200 μl of the culture was spread onto LB plates supplemented with ampicillin (100 μg/ml) and grown overnight at 30 °C. Two independently isolated colonies were picked and inoculated into 5 ml of LB supplemented with ampicillin and grown overnight at 30 °C/250 rpm. These cultures were archived at −80 °C or used for recombineering. Our initial attempts at recombineering using pKD46 were either unsuccessful or yielded very few recombinants. However, allelic replacement was more efficient and reproducible when the lambda red enzymes were expressed from pSIM6. The protocol described herein describes chromosomal manipulations in *E. albertii* using pSIM6 and linear PCR products as substrates.

### PCR Conditions

HiFi Taq DNA Polymerase was used for all PCR reactions essentially as described by the manufacturer (ThermoFisher Scientific). Typically, each PCR was performed in triplicate with each replicate containing 50 μl of the reaction mix. The *cat* and *kan* cassettes were amplified from pKD3 and pKD13 respectively [23]. Briefly, a pair of chimeric primers was used to synthesize the desired dsPCR product to be used in recombineering. Each primer contains two distinct regions: a 3′ region composed of ~19–20 nucleotides that hybridizes to the template (pKD3 or pKD13) and a nonhybridizable 5′ region of ~50 nucleotides in length that is identical to the region immediately upstream or downstream of the locus to be mutated. The PCR reaction yields an amplicon consisting of an FRT-*cat/kan*-FRT cassette that is flanked by two homology arms, each of which is 50 nucleotides in length. A double crossover event between the homology arms and their complementary sequences on the chromosome results in allelic replacement. The conditions for PCR were as follows: [Step 1: 94 °C/5′; Step 2: 94 °C/0.5′; Step 3: 43 °C/0.5′; Step 4: 68 °C/2′; Step 5: Repeat steps 2–4 [x40 cycles]; Step 6: 72 °C/7′; Step 7: 15 °C for ∞]. The synthesis of each amplicon was verified by gel electrophoresis and the PCR product was concentrated using the DNA Clean & Concentrator Kit™-25 from Zymo Research (D4005) into a final volume of 25 μl of elution buffer (10 mM Tris–HCl [pH 8.5], 0.1 mM EDTA). The eluted PCR product was further concentrated by ethanol precipitation. Briefly, 2 μl of glycogen (20 mg/ml), 20 μl of sodium acetate (pH 5.2), and 900 μl of cold 100 % ethanol (−20 °C) were added to the eluted PCR product and thoroughly mixed. The sample was stored at −20 °C for at least 30′ after which it was centrifuged at 4 °C/16000 rpm for 15′. The supernatant was discarded and the pellet washed with 500 μl of 70 % ethanol followed by centrifugation at 4 °C/16000 rpm for 5′ (3x). After the last centrifugation step all the ethanol was vaporized and the sedimented pellet was resuspended in 10 μl of molecular grade water.

### Preparation of Electrocompetent Cells for Recombineering

*E. albertii*(pSIM6) transformants were streak purified onto pre-warmed LB + Amp_100_ and incubated overnight at 30 °C to obtain axenic colonies. A single colony was inoculated into 5 ml of LB + Amp_100_ and incubated ON at 30 °C/250 rpm. The overnight culture was diluted 100 fold in 30 ml of LB + Amp_100_ and grown at 30 °C/250 rpm in an incubator-shaker to an OD_600_ of 0.6–1.0. The cultures were then heat shocked at 42 °C/250 rpm for 1 h in a water bath to induce the expression of the lambda red *exo*, *bet*, and *gam* genes from pSIM6. The pSIM family of plasmids was engineered in the lab of Don Court at the National Institutes of Health (NIH) [28]. Note that we, and other researchers, have observed that recombineering is more efficient when the cultures are thermally induced in a water-shaker as opposed to an air-shaker (data not shown). After induction the cultures were rapidly chilled by swirling in an ice-water bath for ~2′ and then transferred to pre-chilled 50 ml conical tubes. The cultures were then centrifuged at 4100 rpm/4 °C/10′. Subsequently, the supernatant was discarded and each pellet was initially resuspended in 1 ml of ice-cold 10 % glycerol after which the volume was increased to 30 ml by the addition of 29 ml of ice-cold 10 % glycerol. The centrifugation step was repeated and the supernatant discarded. This was followed by suspending the pellet in 1 ml of ice-cold 10 % glycerol and transferring the suspension to a pre-chilled microcentrifuge tube. The tubes were centrifuged at 10,000 rpm/4 °C/2′ and the supernatant discarded. The washes with ice-cold 10 % glycerol were repeated a minimum of 3 times. After the last wash the pellet was resuspended in 400 μl of ice-cold 10 % glycerol. Approximately 90 μl of electrocompetent cells were aliquoted into microcentrifuge tubes and 1.5 μg of the ethanol precipitated PCR product was added to the chilled culture. For the negative control no PCR product was added. The mixture was incubated on ice for ~ 1′ after which it was transferred to a pre-chilled cuvette and electroporation was performed at a voltage of ~1.8–2.5 kV. Immediately after electroporation the cells were recovered in 1 ml of LB and then transferred to a test tube containing 9 ml of LB. The recoverants were incubated at 37 °C/250 rpm for at least 16 h. The next day 1.5 ml of the overnight grown culture was transferred to microcentrifuge tubes and pelleted by centrifugation at 16000 rpm/2′. The supernatant was discarded and the pellet resuspended in ~150–200 μl of LB broth, after which, the cell suspension was plated on LB plates supplemented with chloramphenicol (3.125–25 μg/ml) or kanamycin (15–22.5 μg/ml). The plates were incubated at 37 °C for ~1–2 days until recombinants were observed. Once recombinants arose then individual colonies were inoculated into 50 μl of LB and incubated for ~3 h at 37 °C. Locus-specific PCR was performed on 2 μl of the culture to verify the replacement of the chromosomal allele with the mutant allele.

### Curing of pSIM6

Once recombinants had been identified then ~2 μl of the culture was streaked onto LB+ Cm_6.25_/Kan_22.5_ plates and incubated overnight at 42 °C. The next day a few colonies were picked from each sector and patch plated onto LB + Cm/Kan and LB + Amp plates and incubated overnight at 30 °C. The vast majority of the Cm^R^ or Kan^R^ recombinants were phenotypically Amp^S^ suggesting that the resident plasmid pSIM6 had been cured from the recombinants.

### Eviction of the Drug Resistance Cassette

To evict the *cat* cassette, the recombinants were transformed with the plasmid pFT-K [104]. Transformation was done essentially as described above. pFT-K is a thermolabile replicon that confers temperature-sensitive resistance to kanamycin (25 μg/ml) and expresses the *flp* recombinase under the control of the tetracycline repressor (TetR) of the transposon Tn10*.* All the Kan^R^ transformants that were observed were pooled together into 20 ml of LB supplemented with kanamycin and grown overnight at 30 °C/250 rpm. The overnight grown culture was diluted 100 fold into fresh identical media and incubated at 30 °C under static conditions for 24 h. The following day the FLP recombinase was induced by diluting the overnight culture 4 fold in 20 ml of LB supplemented with unautoclaved chlorotetracycline, at a range of concentrations (20–200 μg/ml), and growing overnight at 30 °C under static conditions. Chlorotetracycline binds to and inactivates the TetR repressor thereby derepressing the *flp* gene. FLP recombinase, in turn, recognizes and catalyzes the site-specific recombination between the two direct repetitive FRT sites to evict the bracketed *cat* resistance cassette, leaving behind a solitary residual FRT site. As a consequence, the engineered bacterial excisant becomes resensitized to chloramphenicol. After overnight induction of the cultures, a loopful of bacteria was streaked onto nonselective LB plates and incubated overnight at 42 °C to cure the thermolabile plasmid. The next day ~36 independently isolated colonies were spot-streaked onto LB, LB + Cm, and LB + Kan and incubated overnight at 30 °C to screen for the Cm^S^ excisants that had also been cured of the plasmid (Kan^S^). If the excisant had not been cured of the plasmid then the Cm^S^ isolate was re-streaked onto fresh LB plates and incubated for another night at 42 °C to cure the plasmid. The genotype of the excisants was further verified by PCR as described above.

## Abbreviations

EPEC: enteropathogenic *Escherichia coli*
EHEC: enterohemorrhagic *Escherichia coli*
EAEC: enteroaggregative *Escherichia coli*
LEE: locus of enterocyte effacement
Ler: LEE encoded regulator
GrlR: global regulator of the LEE repressor
GrlA: global regulator of the LEE activator
Hfq: host factor essential for the replication of the bacteriophage Qß
PCR: polymerase chain reaction
dsDNA: double stranded DNA
ssDNA: single stranded DNA
*eae*: *E. coli* attaching and effacing
rRNA: ribosomal RNA
*stx*: Shiga toxin
ICDDR-B: International Centre for Diarrheal Disease Research, Bangladesh
